# 5-([(*Z*)-Meth­oxy­imino]{2-[(2-methyl­phen­oxy)meth­yl]phen­yl}meth­yl)-1,3,4-oxa­diazole-2(3*H*)-thione dimethyl sulfoxide monosolvate

**DOI:** 10.1107/S2414314623002377

**Published:** 2023-03-28

**Authors:** Chetan Shrimandhar Shripanavar, Rishik Balerao, Ray J. Butcher

**Affiliations:** a Chenshri Research Laboratory (OPC) Private Limited, Pattankudi (Karnataka)-, 591238, India; b Thomas Jefferson High School for Science and Technology, 6560 Braddock Rd, Alexandria VA 22312, USA; c Department of Chemistry, Howard University, 525 College Street NW, Washington DC, 20059, USA; Goethe-Universität Frankfurt, Germany

**Keywords:** crystal structure, DMSO, fungicides, strobilurin

## Abstract

The title compound, C_18_H_17_N_3_O_3_S·C_2_H_6_OS, crystallizes in the monoclinic space group *P*2_1_ */c*. In the crystal, *C*_1_^1^(9) chains of C—H⋯O inter­actions are formed, propogating in the *c*-axis direction. The N—H hydrogen atom forms a strong hydrogen bond with the oxygen atom of a DMSO solvate mol­ecule.

## Abstract 2

The title compound crystallizes in the monoclinic space group *P*2_1_ */c* with one mol­ecule in the asymmetric unit. In the crystal, 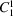 (9) chains of C—H⋯O inter­actions are formed, propogating in the *c*-axis direction. The N—H hydrogen atom forms a strong hydrogen bond with the oxygen atom of a DMSO solvate mol­ecule.

## Graphical abstract

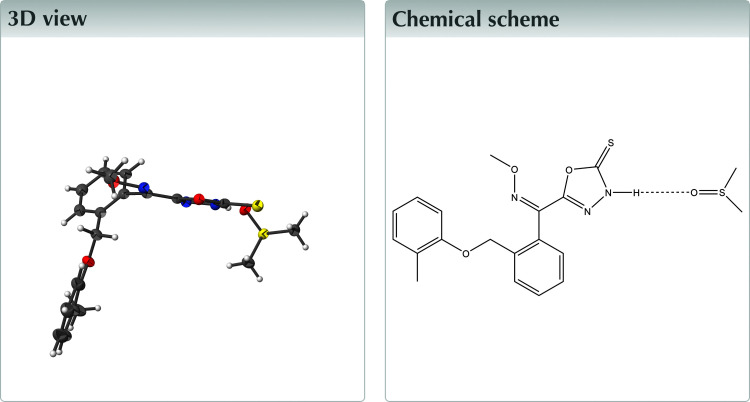

## Structure description

**Figure 1:**
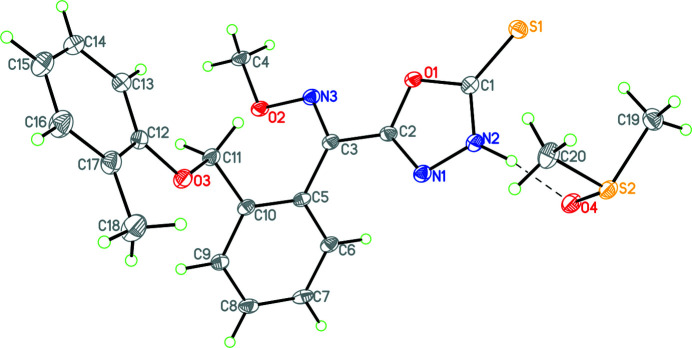
Diagram of **1** showing atom labelling with hydrogen bonding shown as dashed lines and atomic displacement parameters set at the 30% probability level

The newly synthesized title compound C_2_H_6_OS·C_18_H_17_N_3_O_3_S (**1**, Fig. 1 ▸) is derived from Kresoxim methyl fungicide and is a member of the strobilurin family. The broad spectrum nature of **1** allows it to have site-specific action and high efficacy against fungal diseases (Anke *et al.*1977 ▸; Olaya *et al.* 1998 ▸; Patel *et al.*, 2012 ▸; Esteve-Turrillas *et al.*, 2011 ▸; Mercader *et al.*, 2008 ▸; Balba, 2007 ▸; Cash & Cronan, 2001 ▸, Ammermann *et al.*, 2000 ▸; Balba, 2007 ▸; Kant *et al.*, 2012 ▸). The metabolites of compounds such as **1** are easily translocated in nature. The modified structure of **1** has various anti­fungal, anti­bacterial and anti­cancer properties; however, tracing out the exact mode of action of this type of compound will require further study of its bio-efficacy.

Compound **1** crystallizes in the monoclinic space group *P*2_1_ */c* with one strobilurin mol­ecule and one solvent molecule in the asymmetric unit. It consists of a toluene ring linked *via* a meth­oxy group to a phenyl ring, which is then linked to a five-membered 1,3,4 oxa­diazole-2-thione ring. The carbon atom linking the five-membered ring to the phenyl ring additionally has a meth­oxy­amino substituent branching off from it.

**Figure 2:**
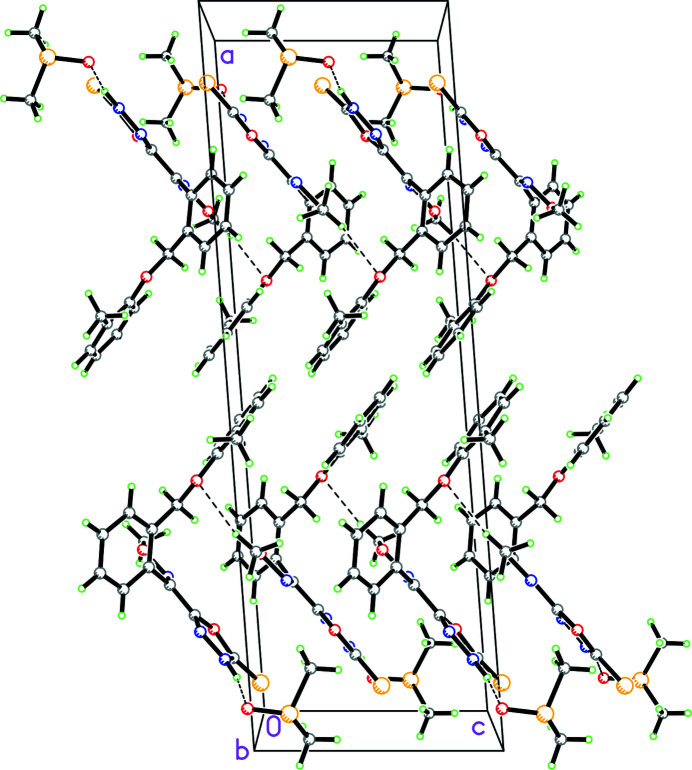
Diagram showing the packing of **1** with hydrogen bonds shown as dashed lines. The mol­ecules of **1** are linked by C—H⋯O inter­actions forming a 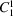 (9) chain propagating in the *c*-axis direction.

In the crystal of **1**, the solvent DMSO mol­ecule accepts both an N—H⋯O hydrogen bond with the five-membered ring and a weak C—H⋯O inter­action with an adjacent DMSO mol­ecule (symmetry operation: *x*, 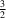 − *y*, − 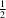 + *z*) (Fig. 2 ▸, Table 1 ▸). Additionally, the meth­oxy­amino substituent forms a weak C—H⋯O inter­action with the meth­oxy group in an adjacent mol­ecule (symmetry operation: *x*, 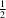 − *y*, 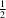 + *z*), forming a 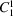 (9) chain propogating in the *c*-axis direction.

A pseudopolymorph of this structure has also be determined with water as solvent (Shripanavar *et al*., 2023 ▸)

## Synthesis and crystallization

The Kresoxim methyl hydrazone compound (3.13 g, 0.01 mol) was dissolved in a solution of KOH (0.01 mol) in water (20 ml) and CS_2_ (0.01 mol) in ethanol (20 ml) and then the mixture was refluxed for 8 h. After the reaction was complete, the mixture was cooled at room temperature and neutralized with diluted HCl (4 *N*). The precipitated product was filtered and washed with water to dry it. Crystals were obtained by evaporation of a DMSO solution.

## Refinement

Crystal data, data collection and structure refinement details for the structure are summarized in Table 2 ▸.

## Supplementary Material

Crystal structure: contains datablock(s) I. DOI: 10.1107/S2414314623002377/bt4133sup1.cif

Structure factors: contains datablock(s) I. DOI: 10.1107/S2414314623002377/bt4133Isup2.hkl

Supporting information file. DOI: 10.1107/S2414314623002377/bt4133Isup3.cml

CCDC reference: 2247969

Additional supporting information:  crystallographic information; 3D view; checkCIF report

## Figures and Tables

**Table 1:** Hydrogen-bond geometry (Å, °)

*D*—H⋯*A*	*D*—H	H⋯*A*	*D*⋯*A*	*D*—H⋯*A*
N2—H2⋯O4	0.99 (5)	1.65 (5)	2.630 (3)	170 (4)
C4—H4*A*⋯O3^i^	0.98	2.54	3.404 (4)	147
C20—H20*C*⋯O4^ii^	0.98	2.45	3.161 (4)	129

Symmetry codes: (i) 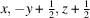 ; (ii) 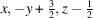 .

**Table 2:** Experimental details

Crystal data	
Chemical formula	C_18_H_17_N_3_O_3_S·C_2_H_6_OS
*M*_r_	433.53
Crystal system, space group	Monoclinic, *P*2_1_/*c*
Temperature (K)	100
*a*, *b*, *c* (Å)	27.0233 (2), 9.00367 (9), 8.96199 (8)
β (°)	94.2689 (8)
*V* (Å^3^)	2174.48 (3)
*Z*	4
Radiation type	Cu *K*α
μ (mm^−1^)	2.48
Crystal size (mm)	0.37 × 0.25 × 0.09

Data collection	
Diffractometer	XtaLAB Synergy, Dualflex, HyPix CCD
Absorption correction	Multi-scan (*CrysAlis PRO*; Rigaku OD, 2022 ▸)
*T*_min_, *T*_max_	0.495, 1.000
No. of measured, independent and observed [*I* > 2σ(*I*)] reflections	79202, 4606, 4232
*R*_int_	0.070
(sin θ/λ)_max_ (Å^−1^)	0.637

Refinement	
*R*[*F* ^2^ > 2σ(*F* ^2^)], *wR*(*F* ^2^), *S*	0.054, 0.166, 1.09
No. of reflections	4606
No. of parameters	270
H-atom treatment	H atoms treated by a mixture of independent and constrained refinement
Δρ_max_, Δρ_min_ (e Å^−3^)	0.92, −0.42

Computer programs: *CrysAlis PRO* (Rigaku OD, 2022 ▸), *SHELXT* (Sheldrick, 2015*b* ▸), *SHELXL2018/3* (Sheldrick, 2015*a* ▸) and *OLEX2* (Dolomanov *et al.*, 2009 ▸).

## full crystallographic data

### Crystal data

**Table:**

C_18_H_17_N_3_O_3_S·C_2_H_6_OS	*F*(000) = 912
*M**_r_* = 433.53	*D*_x_ = 1.324 Mg m^−^^3^
Monoclinic, *P*2_1_/*c*	Cu *K*α radiation, λ = 1.54184 Å
*a* = 27.0233 (2) Å	Cell parameters from 48963 reflections
*b* = 9.00367 (9) Å	θ = 3.3–78.7°
*c* = 8.96199 (8) Å	µ = 2.48 mm^−^^1^
β = 94.2689 (8)°	*T* = 100 K
*V* = 2174.48 (3) Å^3^	Plate, colorless
*Z* = 4	0.37 × 0.25 × 0.09 mm

### Data collection

**Table:**

XtaLAB Synergy, Dualflex, HyPix CCD diffractometer	4232 reflections with *I* > 2σ(*I*)
Radiation source: micro-focus sealed X-ray tube	*R*_int_ = 0.070
ω scans	θ_max_ = 79.0°, θ_min_ = 3.3°
Absorption correction: multi-scan (CrysAlisPro; Rigaku OD, 2022)	*h* = −32→34
*T*_min_ = 0.495, *T*_max_ = 1.000	*k* = −11→9
79202 measured reflections	*l* = −11→11
4606 independent reflections	

### Refinement

**Table:**

Refinement on *F*^2^	0 restraints
Least-squares matrix: full	Hydrogen site location: mixed
*R*[*F*^2^ > 2σ(*F*^2^)] = 0.054	H atoms treated by a mixture of independent and constrained refinement
*wR*(*F*^2^) = 0.166	*w* = 1/[σ^2^(*F*_o_^2^) + (0.074*P*)^2^ + 4.3637*P*] where *P* = (*F*_o_^2^ + 2*F*_c_^2^)/3
*S* = 1.09	(Δ/σ)_max_ = 0.001
4606 reflections	Δρ_max_ = 0.92 e Å^−^^3^
270 parameters	Δρ_min_ = −0.42 e Å^−^^3^

### Special details

**Table:**

Geometry. All esds (except the esd in the dihedral angle between two l.s. planes) are estimated using the full covariance matrix. The cell esds are taken into account individually in the estimation of esds in distances, angles and torsion angles; correlations between esds in cell parameters are only used when they are defined by crystal symmetry. An approximate (isotropic) treatment of cell esds is used for estimating esds involving l.s. planes.
Refinement. A riding model was used for the H atoms attached to C with atomic displacement parameters = 1.2*U*_eq_(C) [1.5*U*_eq_(C) for methyl groups] while the N—H hydrogen atom was refined isotropically.

### Fractional atomic coordinates and isotropic or equivalent isotropic displacement parameters (Å^2^)

**Table:**

	*x*	*y*	*z*	*U*_iso_*/*U*_eq_	
S2	0.93981 (2)	0.70551 (7)	0.33974 (7)	0.02729 (19)	
S1	0.92357 (3)	0.20933 (8)	0.46944 (8)	0.03022 (19)	
O1	0.84852 (7)	0.2364 (2)	0.6449 (2)	0.0247 (4)	
O2	0.74155 (7)	0.1493 (2)	0.9232 (2)	0.0278 (4)	
O3	0.64096 (7)	0.4321 (2)	0.6557 (2)	0.0296 (4)	
O4	0.93495 (8)	0.6691 (2)	0.5039 (2)	0.0323 (5)	
N1	0.84213 (8)	0.4796 (2)	0.6822 (3)	0.0258 (5)	
N2	0.88048 (8)	0.4488 (3)	0.5933 (3)	0.0267 (5)	
H2	0.9011 (17)	0.526 (5)	0.549 (5)	0.069 (14)*	
N3	0.77856 (8)	0.1748 (2)	0.8257 (2)	0.0250 (5)	
C1	0.88506 (10)	0.3029 (3)	0.5679 (3)	0.0253 (5)	
C2	0.82475 (9)	0.3499 (3)	0.7106 (3)	0.0235 (5)	
C3	0.78482 (10)	0.3154 (3)	0.8061 (3)	0.0234 (5)	
C4	0.73450 (11)	−0.0083 (3)	0.9332 (3)	0.0316 (6)	
H4A	0.711008	−0.029760	1.008586	0.047*	
H4B	0.766353	−0.056211	0.961975	0.047*	
H4C	0.721321	−0.046597	0.835861	0.047*	
C5	0.75704 (10)	0.4374 (3)	0.8746 (3)	0.0234 (5)	
C6	0.78357 (10)	0.5348 (3)	0.9721 (3)	0.0265 (5)	
H6	0.818646	0.526881	0.986990	0.032*	
C7	0.75861 (11)	0.6433 (3)	1.0473 (3)	0.0296 (6)	
H7	0.776578	0.708605	1.114703	0.036*	
C8	0.70765 (11)	0.6562 (3)	1.0241 (3)	0.0304 (6)	
H8	0.690546	0.729492	1.076587	0.036*	
C9	0.68135 (10)	0.5619 (3)	0.9237 (3)	0.0281 (6)	
H9	0.646474	0.573439	0.905669	0.034*	
C10	0.70567 (10)	0.4506 (3)	0.8493 (3)	0.0248 (5)	
C11	0.67522 (10)	0.3450 (3)	0.7495 (3)	0.0275 (6)	
H11A	0.697051	0.286843	0.687417	0.033*	
H11B	0.656964	0.275053	0.810603	0.033*	
C12	0.60536 (10)	0.3565 (3)	0.5680 (3)	0.0281 (6)	
C13	0.59919 (11)	0.2033 (3)	0.5694 (4)	0.0331 (6)	
H13	0.620853	0.142935	0.631872	0.040*	
C14	0.56100 (12)	0.1388 (4)	0.4785 (4)	0.0398 (7)	
H14	0.556604	0.034137	0.479031	0.048*	
C15	0.52973 (13)	0.2257 (4)	0.3882 (4)	0.0468 (8)	
H15	0.503516	0.181535	0.326974	0.056*	
C16	0.53654 (13)	0.3779 (4)	0.3866 (4)	0.0458 (8)	
H16	0.514667	0.437152	0.323659	0.055*	
C17	0.57434 (11)	0.4464 (4)	0.4742 (4)	0.0353 (6)	
C18	0.58288 (13)	0.6118 (4)	0.4704 (5)	0.0477 (9)	
H18A	0.555770	0.659212	0.409096	0.072*	
H18B	0.614445	0.632245	0.427202	0.072*	
H18C	0.583968	0.651520	0.572391	0.072*	
C19	0.98058 (11)	0.5696 (4)	0.2734 (3)	0.0349 (6)	
H19A	1.014221	0.586410	0.318900	0.052*	
H19B	0.980684	0.577262	0.164293	0.052*	
H19C	0.969320	0.470396	0.300279	0.052*	
C20	0.88338 (11)	0.6408 (4)	0.2451 (4)	0.0385 (7)	
H20A	0.855370	0.697025	0.279692	0.058*	
H20B	0.879052	0.535046	0.266733	0.058*	
H20C	0.884759	0.654678	0.137066	0.058*	

### Atomic displacement parameters (Å^2^)

**Table:**

	*U*^11^	*U*^22^	*U*^33^	*U*^12^	*U*^13^	*U*^23^
S2	0.0319 (4)	0.0229 (3)	0.0273 (3)	−0.0070 (2)	0.0040 (3)	0.0002 (2)
S1	0.0311 (4)	0.0309 (4)	0.0289 (4)	0.0015 (3)	0.0036 (3)	−0.0026 (3)
O1	0.0276 (9)	0.0187 (9)	0.0277 (9)	−0.0018 (7)	0.0025 (7)	−0.0005 (7)
O2	0.0350 (10)	0.0162 (9)	0.0332 (10)	−0.0029 (7)	0.0085 (8)	0.0020 (7)
O3	0.0259 (9)	0.0240 (10)	0.0378 (11)	−0.0017 (7)	−0.0040 (8)	0.0012 (8)
O4	0.0410 (11)	0.0297 (10)	0.0268 (10)	−0.0105 (8)	0.0069 (8)	−0.0031 (8)
N1	0.0278 (11)	0.0208 (11)	0.0287 (11)	−0.0013 (8)	0.0021 (9)	0.0036 (9)
N2	0.0280 (11)	0.0239 (11)	0.0285 (11)	−0.0031 (9)	0.0041 (9)	0.0016 (9)
N3	0.0285 (11)	0.0208 (10)	0.0256 (11)	−0.0022 (8)	0.0029 (9)	0.0009 (9)
C1	0.0274 (13)	0.0249 (13)	0.0231 (12)	−0.0021 (10)	−0.0012 (10)	0.0023 (10)
C2	0.0265 (12)	0.0181 (12)	0.0254 (12)	0.0014 (9)	−0.0006 (10)	0.0002 (9)
C3	0.0257 (12)	0.0186 (12)	0.0253 (12)	−0.0014 (9)	−0.0016 (10)	0.0013 (9)
C4	0.0433 (16)	0.0171 (12)	0.0352 (15)	−0.0072 (11)	0.0087 (12)	−0.0006 (11)
C5	0.0303 (13)	0.0137 (11)	0.0262 (12)	−0.0012 (9)	0.0015 (10)	0.0037 (9)
C6	0.0304 (13)	0.0182 (12)	0.0300 (13)	−0.0013 (10)	−0.0034 (10)	0.0047 (10)
C7	0.0413 (15)	0.0161 (12)	0.0305 (14)	−0.0011 (10)	−0.0039 (11)	0.0015 (10)
C8	0.0401 (15)	0.0153 (12)	0.0360 (15)	0.0011 (11)	0.0048 (12)	0.0029 (10)
C9	0.0299 (13)	0.0187 (12)	0.0359 (14)	0.0002 (10)	0.0035 (11)	0.0028 (10)
C10	0.0295 (13)	0.0165 (12)	0.0284 (13)	−0.0030 (9)	0.0019 (10)	0.0036 (10)
C11	0.0259 (13)	0.0206 (12)	0.0355 (14)	−0.0011 (10)	−0.0008 (11)	0.0019 (11)
C12	0.0237 (12)	0.0306 (14)	0.0300 (13)	−0.0025 (10)	0.0027 (10)	−0.0047 (11)
C13	0.0292 (14)	0.0316 (15)	0.0384 (16)	−0.0012 (11)	0.0025 (12)	−0.0067 (12)
C14	0.0343 (15)	0.0355 (17)	0.0497 (19)	−0.0050 (12)	0.0035 (13)	−0.0137 (14)
C15	0.0359 (17)	0.051 (2)	0.051 (2)	−0.0069 (15)	−0.0074 (14)	−0.0135 (16)
C16	0.0384 (17)	0.049 (2)	0.0479 (19)	−0.0031 (15)	−0.0100 (14)	−0.0001 (16)
C17	0.0302 (14)	0.0368 (16)	0.0382 (16)	−0.0017 (12)	−0.0018 (12)	0.0016 (13)
C18	0.0419 (18)	0.0383 (18)	0.060 (2)	−0.0029 (14)	−0.0146 (15)	0.0120 (16)
C19	0.0339 (15)	0.0394 (17)	0.0316 (14)	−0.0002 (12)	0.0049 (11)	−0.0022 (12)
C20	0.0313 (15)	0.0426 (18)	0.0406 (17)	−0.0074 (13)	−0.0038 (12)	0.0057 (14)

### Geometric parameters (Å, º)

**Table:**

S2—O4	1.523 (2)	C8—C9	1.393 (4)
S2—C19	1.778 (3)	C9—H9	0.9500
S2—C20	1.787 (3)	C9—C10	1.394 (4)
S1—C1	1.645 (3)	C10—C11	1.508 (4)
O1—C1	1.382 (3)	C11—H11A	0.9900
O1—C2	1.364 (3)	C11—H11B	0.9900
O2—N3	1.395 (3)	C12—C13	1.390 (4)
O2—C4	1.436 (3)	C12—C17	1.400 (4)
O3—C11	1.436 (3)	C13—H13	0.9500
O3—C12	1.376 (3)	C13—C14	1.393 (4)
N1—N2	1.382 (3)	C14—H14	0.9500
N1—C2	1.291 (3)	C14—C15	1.371 (5)
N2—H2	0.99 (5)	C15—H15	0.9500
N2—C1	1.340 (4)	C15—C16	1.383 (5)
N3—C3	1.291 (3)	C16—H16	0.9500
C2—C3	1.460 (4)	C16—C17	1.386 (4)
C3—C5	1.488 (4)	C17—C18	1.508 (5)
C4—H4A	0.9800	C18—H18A	0.9800
C4—H4B	0.9800	C18—H18B	0.9800
C4—H4C	0.9800	C18—H18C	0.9800
C5—C6	1.398 (4)	C19—H19A	0.9800
C5—C10	1.395 (4)	C19—H19B	0.9800
C6—H6	0.9500	C19—H19C	0.9800
C6—C7	1.389 (4)	C20—H20A	0.9800
C7—H7	0.9500	C20—H20B	0.9800
C7—C8	1.382 (4)	C20—H20C	0.9800
C8—H8	0.9500		
			
O4—S2—C19	105.99 (14)	C9—C10—C11	118.8 (2)
O4—S2—C20	104.85 (13)	O3—C11—C10	107.6 (2)
C19—S2—C20	98.34 (16)	O3—C11—H11A	110.2
C2—O1—C1	105.6 (2)	O3—C11—H11B	110.2
N3—O2—C4	107.8 (2)	C10—C11—H11A	110.2
C12—O3—C11	117.2 (2)	C10—C11—H11B	110.2
C2—N1—N2	103.4 (2)	H11A—C11—H11B	108.5
N1—N2—H2	124 (3)	O3—C12—C13	124.4 (3)
C1—N2—N1	112.2 (2)	O3—C12—C17	114.8 (3)
C1—N2—H2	124 (3)	C13—C12—C17	120.8 (3)
C3—N3—O2	110.7 (2)	C12—C13—H13	120.3
O1—C1—S1	123.4 (2)	C12—C13—C14	119.5 (3)
N2—C1—S1	131.5 (2)	C14—C13—H13	120.3
N2—C1—O1	105.1 (2)	C13—C14—H14	119.8
O1—C2—C3	119.1 (2)	C15—C14—C13	120.3 (3)
N1—C2—O1	113.7 (2)	C15—C14—H14	119.8
N1—C2—C3	127.1 (2)	C14—C15—H15	120.2
N3—C3—C2	113.5 (2)	C14—C15—C16	119.7 (3)
N3—C3—C5	126.3 (2)	C16—C15—H15	120.2
C2—C3—C5	120.1 (2)	C15—C16—H16	119.0
O2—C4—H4A	109.5	C15—C16—C17	121.9 (3)
O2—C4—H4B	109.5	C17—C16—H16	119.0
O2—C4—H4C	109.5	C12—C17—C18	119.9 (3)
H4A—C4—H4B	109.5	C16—C17—C12	117.7 (3)
H4A—C4—H4C	109.5	C16—C17—C18	122.3 (3)
H4B—C4—H4C	109.5	C17—C18—H18A	109.5
C6—C5—C3	118.0 (2)	C17—C18—H18B	109.5
C10—C5—C3	121.5 (2)	C17—C18—H18C	109.5
C10—C5—C6	120.4 (2)	H18A—C18—H18B	109.5
C5—C6—H6	120.0	H18A—C18—H18C	109.5
C7—C6—C5	120.0 (3)	H18B—C18—H18C	109.5
C7—C6—H6	120.0	S2—C19—H19A	109.5
C6—C7—H7	120.0	S2—C19—H19B	109.5
C8—C7—C6	120.0 (3)	S2—C19—H19C	109.5
C8—C7—H7	120.0	H19A—C19—H19B	109.5
C7—C8—H8	120.0	H19A—C19—H19C	109.5
C7—C8—C9	120.0 (3)	H19B—C19—H19C	109.5
C9—C8—H8	120.0	S2—C20—H20A	109.5
C8—C9—H9	119.6	S2—C20—H20B	109.5
C8—C9—C10	120.7 (3)	S2—C20—H20C	109.5
C10—C9—H9	119.6	H20A—C20—H20B	109.5
C5—C10—C11	122.4 (2)	H20A—C20—H20C	109.5
C9—C10—C5	118.8 (2)	H20B—C20—H20C	109.5
			
O1—C2—C3—N3	4.4 (3)	C3—C5—C10—C11	−1.2 (4)
O1—C2—C3—C5	−177.7 (2)	C4—O2—N3—C3	176.7 (2)
O2—N3—C3—C2	177.3 (2)	C5—C6—C7—C8	−0.9 (4)
O2—N3—C3—C5	−0.5 (4)	C5—C10—C11—O3	−135.1 (2)
O3—C12—C13—C14	178.3 (3)	C6—C5—C10—C9	−0.4 (4)
O3—C12—C17—C16	−177.8 (3)	C6—C5—C10—C11	−177.8 (2)
O3—C12—C17—C18	2.5 (4)	C6—C7—C8—C9	−0.9 (4)
N1—N2—C1—S1	179.2 (2)	C7—C8—C9—C10	2.1 (4)
N1—N2—C1—O1	−0.7 (3)	C8—C9—C10—C5	−1.4 (4)
N1—C2—C3—N3	−173.5 (3)	C8—C9—C10—C11	176.1 (2)
N1—C2—C3—C5	4.4 (4)	C9—C10—C11—O3	47.5 (3)
N2—N1—C2—O1	−0.9 (3)	C10—C5—C6—C7	1.6 (4)
N2—N1—C2—C3	177.1 (2)	C11—O3—C12—C13	2.9 (4)
N3—C3—C5—C6	116.6 (3)	C11—O3—C12—C17	−177.4 (2)
N3—C3—C5—C10	−60.1 (4)	C12—O3—C11—C10	−172.3 (2)
C1—O1—C2—N1	0.5 (3)	C12—C13—C14—C15	0.0 (5)
C1—O1—C2—C3	−177.6 (2)	C13—C12—C17—C16	1.9 (5)
C2—O1—C1—S1	−179.72 (19)	C13—C12—C17—C18	−177.8 (3)
C2—O1—C1—N2	0.1 (3)	C13—C14—C15—C16	0.6 (5)
C2—N1—N2—C1	1.0 (3)	C14—C15—C16—C17	0.0 (6)
C2—C3—C5—C6	−61.1 (3)	C15—C16—C17—C12	−1.3 (5)
C2—C3—C5—C10	122.3 (3)	C15—C16—C17—C18	178.4 (4)
C3—C5—C6—C7	−175.1 (2)	C17—C12—C13—C14	−1.3 (4)
C3—C5—C10—C9	176.2 (2)		

### Hydrogen-bond geometry (Å, º)

**Table:**

*D*—H···*A*	*D*—H	H···*A*	*D*···*A*	*D*—H···*A*
N2—H2···O4	0.99 (5)	1.65 (5)	2.630 (3)	170 (4)
C4—H4*A*···O3^i^	0.98	2.54	3.404 (4)	147
C11—H11*A*···N3	0.99	2.65	3.214 (3)	117
C20—H20*C*···O4^ii^	0.98	2.45	3.161 (4)	129

Symmetry codes: (i) *x*, −*y*+1/2, *z*+1/2; (ii) *x*, −*y*+3/2, *z*−1/2.
